# Utility of a smartphone based system (cvrphone) to accurately determine apneic events from electrocardiographic signals

**DOI:** 10.1371/journal.pone.0217217

**Published:** 2019-06-17

**Authors:** Kwanghyun Sohn, Faisal M. Merchant, Shady Abohashem, Kanchan Kulkarni, Jagmeet P. Singh, E. Kevin Heist, Chris Owen, Jesse D. Roberts, Eric M. Isselbacher, Furrukh Sana, Antonis A. Armoundas

**Affiliations:** 1 Cardiovascular Research Center, Massachusetts General Hospital, Boston, MA, United States of America; 2 Cardiology Division, Emory University School of Medicine, Atlanta, GA, United States of America; 3 Cardiology Division, Cardiac Arrhythmia Service, Massachusetts General Hospital, Boston, MA, United States of America; 4 Neurosurgery Division, Massachusetts General Hospital, Boston, MA, United States of America; 5 Anesthesia, Critical Care and Pain Medicine, Massachusetts General Hospital, Boston, MA, United States of America; 6 Healthcare Transformation Lab, Massachusetts General Hospital, Boston, MA, United States of America; 7 Institute for Medical Engineering and Science, Massachusetts Institute of Technology Cambridge, MA, United States of America; Kaplan Medical Center, ISRAEL

## Abstract

**Background:**

Sleep disordered breathing manifested as sleep apnea (SA) is prevalent in the general population, and while it is associated with increased morbidity and mortality risk in some patient populations, it remains under-diagnosed. The objective of this study was to assess the accuracy of respiration-rate (RR) and tidal-volume (TV) estimation algorithms, from body-surface ECG signals, using a smartphone based ambulatory respiration monitoring system (cvrPhone).

**Methods:**

Twelve lead ECG signals were collected using the cvrPhone from anesthetized and mechanically ventilated swine (n = 9). During ECG data acquisition, the mechanical ventilator tidal-volume (TV) was varied from 250 to 0 to 750 to 0 to 500 to 0 to 750 ml at respiratory rates (RR) of 6 and 14 breaths/min, respectively, and the RR and TV values were estimated from the ECG signals using custom algorithms.

**Results:**

TV estimations from any two different TV settings showed statistically significant difference (p < 0.01) regardless of the RR. RRs were estimated to be 6.1±1.1 and 14.0±0.2 breaths/min at 6 and 14 breaths/min, respectively (when 250, 500 and 750 ml TV settings were combined). During apnea, the estimated TV and RR values were 11.7±54.9 ml and 0.0±3.5 breaths/min, which were significantly different (p<0.05) than TV and RR values during non-apnea breathing. In addition, the time delay from the apnea onset to the first apnea detection was 8.6±6.7 and 7.0±3.2 seconds for TV and RR respectively.

**Conclusions:**

We have demonstrated that apnea can reliably be detected using ECG-derived RR and TV algorithms. These results support the concept that our algorithms can be utilized to detect SA in conjunction with ECG monitoring.

## Introduction

Respiration rate (RR) and tidal volume (TV) monitoring are an essential component of patient care in emergency rooms, intensive care units and they are employed during mechanical ventilation of patients with acute lung injury, acute respiratory distress syndrome, etc. [1], [2]. RR and/or TV can be measured using a number of different methods, such as spirometer [3], Pitot tube [4], respiratory inductance plethysmography [5], impedance plethysmography [6], and computed tomography [7]. In the clinical setting, specialized hardware employing these standard techniques provide efficient measurement of RR and TV, in ambulatory setting their bulkiness often makes patient monitoring of these parameters, impractical.

Recent advances in hardware technology and software processing algorithms have enabled the development of much smaller, light-weight, and reliable systems for ambulatory cardiorespiratory monitoring [8]. These systems can now include standalone wearable and implantable sensor devices [9] that can be integrated with an application (app) [10], [11] for acquisition, processing, and monitoring of the data. For ECG monitoring, for example, the ZioPatch cardiac monitor is a device that utilizes a single-lead adhesive chest patch for continuous long-term ECG monitoring. A clinical study, showed that ZioPatch was more effective in detecting arrhythmias than the traditional Holter monitor [9]. Another wearable device called the CoVa necklace can monitor the heart rate, heart rate variability, thoracic fluid index, and respiration rate [12]. AliveCor’s Kardia Mobile (KM) is an ECG event recorder that uses a smartphone app for ambulatory monitoring. A study that compared KM to an external loop recorder (ELR) for clinical diagnosis, found that the KM had the potential to provide a better diagnosis of cardiac events, such as increased heart rate or atrial fibrillation, than the ELR (100% vs. 72.7%) [10]. Apple Inc. has also incorporated ECG monitoring functionality in its iWatch devices. These devices have been reported to have a good sensitivity (87%) and specificity (97%) when detecting silent atrial fibrillation [11]. Non-invasive techniques targeting ambulatory monitoring of RR and TV have also been developed that have resulted in portable devices incorporated into garments [13], [14], [15]. Although these systems incorporate algorithms that are optimized to record and interpret ECG signals, it is desirable to develop advanced signal processing methods that can utilize these cardiac signals to measure and analyze other physiological parameters.

In the studies detailed here, we tested a novel method that extracts RR and TV information in real-time from ECG data, based on the observation that repetitious inflation and deflation of the lungs causes oscillations in the heart position, the electrode locations, and the thoracic impedance. Accordingly, we have hypothesized that respiratory signals may be obtained by measuring fluctuations in the mean cardiac electrical axis [1], [2], [16]. Previously, we developed novel algorithms that extract a respiratory signal from ECG signals using the root-mean-squared amplitude of the QRS complex on a beat-by-beat basis, and thereby permit the estimation of the RR [1] and TV [2]. We have also developed a novel smartphone based ambulatory cardiac and respiratory monitoring system and developed applications (apps) to estimate the RR and TV [17]. In this study, we tested the hypothesis that 12-lead ECG signals processed by a novel mobile cvrPhone system could detect apnea using a mechanically ventilated animal model.

## Methods

### Animal preparation and data recording

The animal studies were approved by the institutional review board and the Subcommittee on Research Animal Care at the Massachusetts General Hospital. All experiments were performed in accordance with relevant guidelines and regulations.

Nine male Yorkshire swine were anesthetized and instrumented in the Animal Electrophysiology Laboratory at Massachusetts General Hospital, as previously described [1], [2]. Anesthesia was induced with Telazol (4.4 mg/kg) IM. Each animal was intubated and placed on a mechanical ventilator and anesthesia was maintained with Isoflurane (1.5–2.5%) and 2% O_2_. Paralytics were used to control spontaneous of the thoracic cavity during apnea. The system incorporated a volume-controlled, time-cycled ventilator (Ohmeda-GE, Madison, WI) and a capnograph (Surgivet V9004, Smiths Medical, Dublin, OH), which was used to confirm the RR delivered by the ventilator throughout the respiratory interventions. The capnograph monitor has an accuracy of ±1 breath/min. Electrodes were placed at the standard 12-lead ECG placement locations.

**Fig 1:**
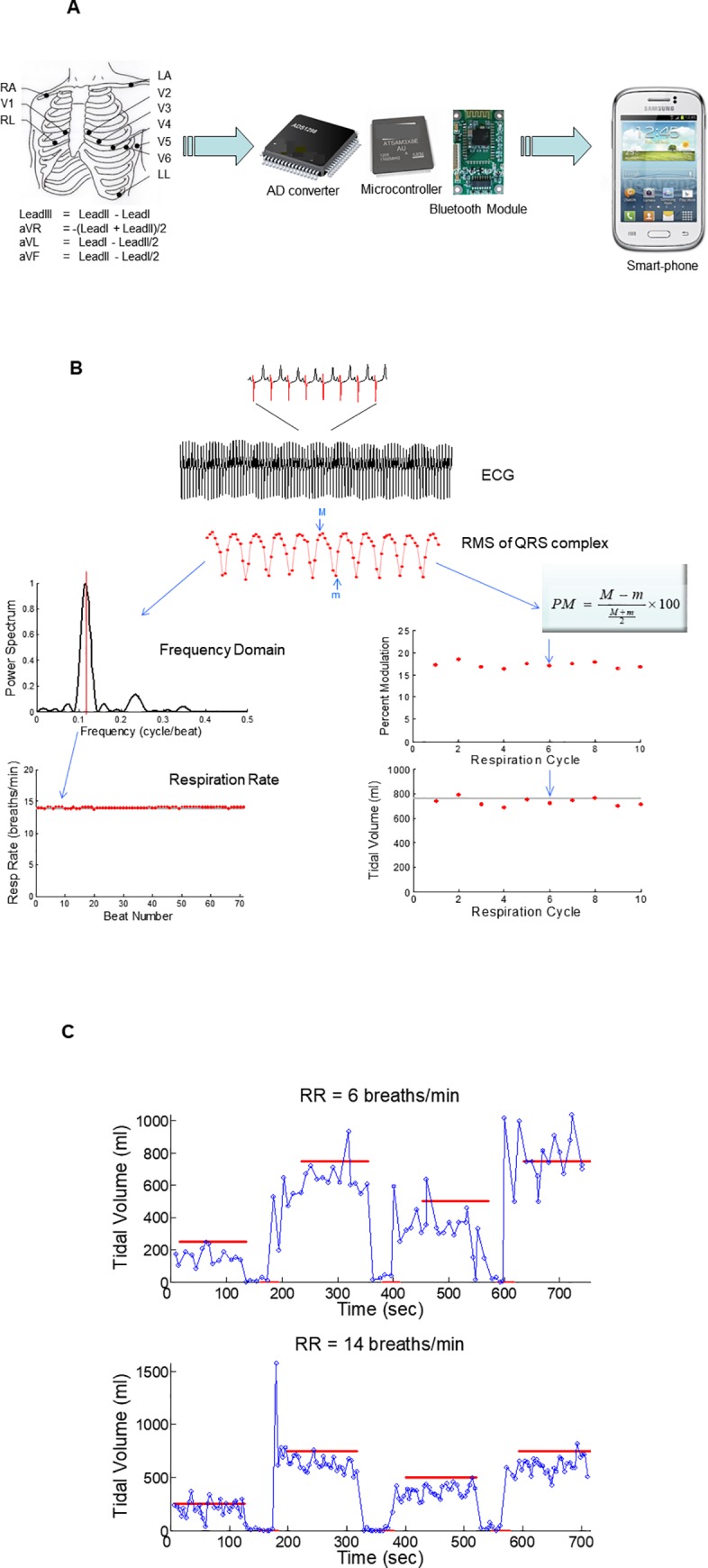
(A) The Smart-Phone based ECG acquisition system, cvrPhone. Flow-diagram of the 12-lead ECG signals from the torso to Smart-Phone. Ten electrodes are placed on the torso for the recording of 8 ECG leads (Leads I and II and six precordial leads) and the real-time display of selected three ECG signals on the smartphone screen. (Modified from [17] under a CC BY license, with permission from the authors, original copyright 2017). (B) Lay-out of the algorithms to estimate the RR and TV. (C) Estimation of the tidal-volume (TV) at 250 to 0 to 750 to 0 to 500 to 0 to 750 ml (marked by the red lines), at RR of 6 breaths/min (upper panel) and 14 breaths/min (lower panel) set by adjusting the mechanical ventilator and using the value indicated by the capnogragh monitor as the gold standard.

Studies were performed while the animal was in a supine position and the RR was set at 6 or 14 breaths/min and the TV was changed from 250 to 0 to 750 to 0 to 500 to 0 to 750 ml, for 2 min at each level. During the apnea period the ventilator was suspended for 30 sec; this period of time was chosen to replicate the duration of a typical apneic event. ECG signals were recorded using the cvrPhone (Fig 1) as previously reported [17]. The analog signals were digitized at 500 Hz and 16 bit resolution (~0.38 μV). The Wilson Central Terminal (WCT, (RA+LA+LL)/3) was used as the reference voltage for the precordial leads.

### ECG-processing algorithms

A software-based QRS detection algorithm was applied, to a predetermined lead, to obtain preliminary R-wave annotations. The preliminary QRS detections were refined, and abnormal beats, e.g., premature ventricular complexes and aberrantly conducted beats, were identified using a template-matching QRS alignment algorithm [1], [2]. Briefly, for each new beat an 80 ms window centered at the peak of the QRS complex was formed from the preliminary beat detection, and an isoelectric PR segment was automatically subtracted as a zero amplitude reference point (by estimating the mean voltage in a 10 ms window preceding the start of each QRS complex). A median QRS template was generated from all normal QRS complexes across the previous 31 beats and the beat was aligned to the QRS template using cross-correlation. Cross-correlation was repeated twice for each new QRS complex to ensure proper QRS alignment. A beat was considered abnormal if its correlation coefficient was less than a threshold value of 0.90, or if the preceding R-to-R interval was at least 10% shorter than the mean RR interval of the previous 7 beats.

An overall description of the algorithms to estimate the RR and TV are presented in Fig 1B. To extract the respiration-induced periodic modulation of the ECG signals, we estimated, on a beat-by-beat basis, the root mean square (RMS) value of the ECG signal in a 100 ms window centered at the peak of the QRS complex. The derived RMS envelope exhibited periodic oscillation [1], [2], [17].

### Respiration-rate estimation algorithm

Then, the respirophasic signal for lead pairs was calculated as the RMS signal ratio on a beat-by-beat basis. Specifically, each lead pair combination consisted of a test lead (the numerator), and a reference lead (the denominator). For each ECG lead pair combination we calculated the respirophasic signal as the 32 beat length RMS signal ratio on a beat-by-beat basis. Thereafter, we estimated the power spectrum of the RMS ratio data using a 512-length Fourier transform to improve the frequency-domain resolution. The dominant power spectral peak between 0.03 and 0.3 cycles/beat was detected, and the signal-to-noise ratio (SNR) was calculated, as the spectral peak power divided by the mean of the power spectrum from 0 to 0.5 cycles/beat, expressed in decibels:

$$SNR=10\log_{10}\left(\frac{signal}{noise}\right)$$

We calculated SNR values for every combination of lead pairs, and selected the pair with highest SNR for RR estimation across all 144 permutations. The selected peak frequency in cycles/beat was converted to breaths per minute by scaling the frequency by the average heart-rate (HR) across the 32 beat window. If there were more than 10% abnormal beats in the 32 beat window, then the corresponding RR was linearly interpolated. A frequency in the Fast Fourier Transform spectrum that is smaller than 0.03 cycles per heart beat was considered to be an apnea event, and zero was assigned to the corresponding RR estimation.

### Tidal-volume estimation algorithm

The TV was estimated using the peak-to-peak amplitude of the respiratory RMS signal, as we detailed previously [2]. To account for cases of high RR and low HR, which could effect the accuracy of the peak-to-peak estimation of the RMS signal, we used cubic spline interpolation to double the number of the RMS signal samples. Subsequently, the peak-to-peak amplitude of the respiratory envelope was normalized to the mean value to obtain the percent modulation (PM): 100× (max envelope–min envelope)/(max envelope + min envelope)/2. [2]

Then, we found the median PM from all leads in a running 10 seconds window, and applied a regression equation (TV = 45.65 × PM -51.15) on the median PM to estimate the corresponding TV [2]. When the estimated TV is negative, the value is set to zero.

### Statistics

Our results are presented as median ± standard deviation of normally distributed variables, unless otherwise noted. The Wilcoxon rank sum test was used to compare two related samples. A statistically significant change is manifested by a p < 0.05. Statistical analysis was performed using MATLAB (MathWorks Inc, Natick, MA).

## Results

The RR and TV estimation depends on the HR (the HR corresponds to the sampling rates for the TV and RR estimation algorithms). In this study, the HR was 114±11 beats per minute.

### Apnea detection using tidal-volume

To assess the ability of our TV estimation algorithm to detect apnea, short respiratory pauses were induced in anesthetized pigs using a mechanical ventilator. The respiratory pause duration was chosen to replicate clinically relevant periods of apnea. Each transition in the respiratory rate was confirmed by reading the target TV on the mechanical ventilator display. Fig 1C shows examples of the time-dependent trace of TV estimation values at RR of 6 breaths/min (upper panel) and 14 breaths/min (lower panel). Each red line of Fig 1C represents the time when the target TV setting was displayed at the mechanical ventilator. The target setting was maintained for 30 seconds during apnea episodes and for 2 minutes during every other TV setting episodes. Each circle in Fig 1C represents the timing of the positive peak and the estimated TV.

**Fig 2:**
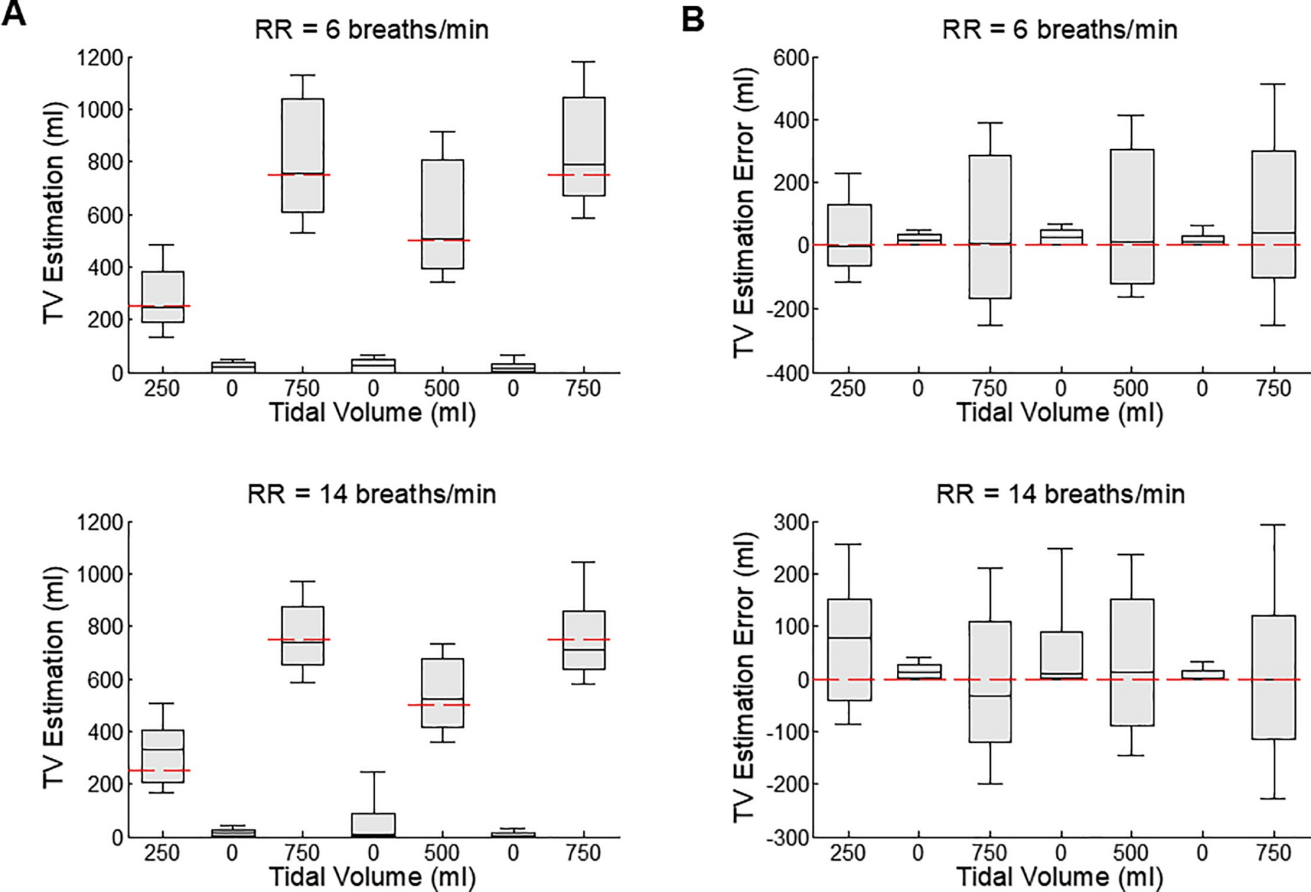
Tidal volume estimations (A) and the estimation errors (B), in the order of the tidal volume setting. The tidal volume setting of the mechanical ventilator was changed from 250 to 0 to 750 to 0 to 500 to 0 to 750 ml, as marked by the red thick lines on (A). The respiration rate was 6 breaths/min for the upper two panels and 14 breaths/min for the lower two panels. There were 9 records from 9 animals in case of 6 breaths/min, and 8 records from 8 animals in case of 14 breaths/min. Each bar plot represents 90, 75, 50, 25 & 10% of all estimated values (A) or of all estimation errors (B). There was difference in the estimated TV errors between any two apneic events or between the two 750 ml settings. On the other hand, the TV estimation error was between 0 and 250 ml (p = 0.34, p = 0.23 & p = 0.29 at 6 breaths/min, and p<0.05, p = 0.66 & p<0.05 at 14 breaths/min), 250 and 500 ml (p = 0.54 at 6 breaths/min, and p<0.05 at 14 breaths/min), and 500 and 750 ml (p = 0.15 & p = 0.62 at 6 breaths/min, and p<0.05 & p = 0.16 at 14 breaths/min).

Fig 2A shows TV estimations at the indicated mechanical ventilation rates (9 animals at 6 breaths/min RR and from 8 animals at 14 breaths/min RR). Again, the red dashed lines indicate the TV settings of the ventilator. For every pair of adjacent TV settings, there is a difference between the two groups of estimated TV values (p<0.05). Estimated TV values during apnea are distinguished from adjacent non-apnea settings. There was no difference in the estimated TV errors between any two apneic events or between the two 750 ml settings (Fig 2B). On the other hand, the TV estimation error was between 0 and 250 ml (p = 0.34, p = 0.23 & p = 0.29 at 6 breaths/min, and p<0.05, p = 0.66 & p<0.05 at 14 breaths/min), 250 and 500 ml (p = 0.54 at 6 breaths/min, and p<0.05 at 14 breaths/min), and 500 and 750 ml (p = 0.15 & p = 0.62 at 6 breaths/min, and p<0.05 & p = 0.16 at 14 breaths/min).

**Fig 3:**
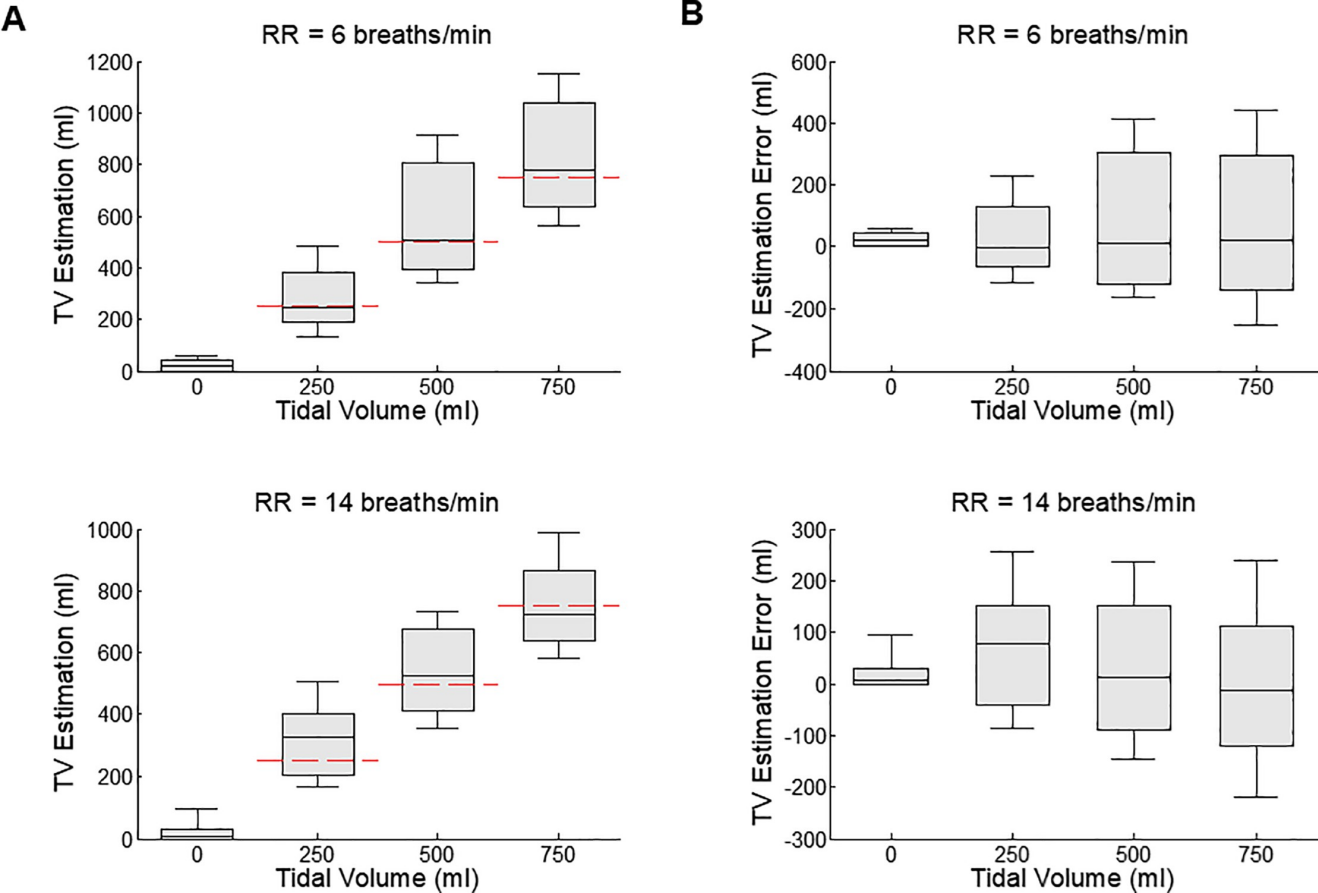
Tidal volume (TV) estimations (A) and TV estimation errors (B), in the order of increasing TV. The tidal volume setting of the mechanical ventilator was changed from 250 to 0 to 750 to 0 to 500 to 0 to 750 ml during tests, and rearranged in the order of increasing TV in the plots. The TV setting values are marked by the red thick lines on the left panels. The respiration rate was 6 breaths/min for the upper two panels and 14 breaths/min for the lower two panels. There were 9 records from 9 animals in case of 6 breaths/min, and 8 records from 8 animals in case of 14 breaths/min. Each bar plot represents 90, 75, 50, 25 & 10% of all estimated values (A) or estimation errors (B). All pairs of adjacent TVs in (A) are different with 0 vs 250 ml (p<0.001), 250 vs 500 ml (p<0.001) and 500 vs 750 ml (p<0.001) for 6 breaths/min and 0 vs 250 ml (p< = 0.001), 250 vs 500 ml (p<0.001) and 500 vs 750 ml (p<0.001) for 14 breaths/min. In (B), the TV estimation errors increase with incremental TV values, and the magnitude of these errors at 6 breaths/min is larger (p = 0.61, p = 0.89 & p = 0.07 at 250, 500 & 750 ml, respectively) than the errors at 14 breaths/min during non-apnea events.

Fig 3A shows TV estimations arranged in the order of increasing true TV values from 0 to 750 ml. The median estimated TV values are 20, 249, 506, and 774 ml at 6 breaths/min RR, and 7, 328, 522, and 720 ml at 14 breaths/min RR at 0, 250, 500 and 750 ml respectively. All pairs of adjacent TVs are significantly different, with 0 vs 250 ml (p<0.001), 250 vs 500 ml (p<0.001) and 500 vs 750 ml (p<0.001) for 6 breaths/min and 0 vs 250 ml (p<0.001), 250 vs 500 ml (p<0.001) and 500 vs 750 ml (p<0.001) for 14 breaths/min. Linear regression between the estimated and true TV showed an R of 0.7877 (p<0.0001) at 6 breaths/min and 0.8233 (p<0.0001) at 14 breaths/min. In Fig 3B, we determined that the TV estimation errors increase with incremental TV values, and the magnitude of these errors at 6 breaths/min is larger (p = 0.61, 0.89 & 0.07 at 250, 500 & 750 ml, respectively) than the errors at 14 breaths/min during non-apnea events.

### Apnea detection using respiration-rate

**Fig 4:**
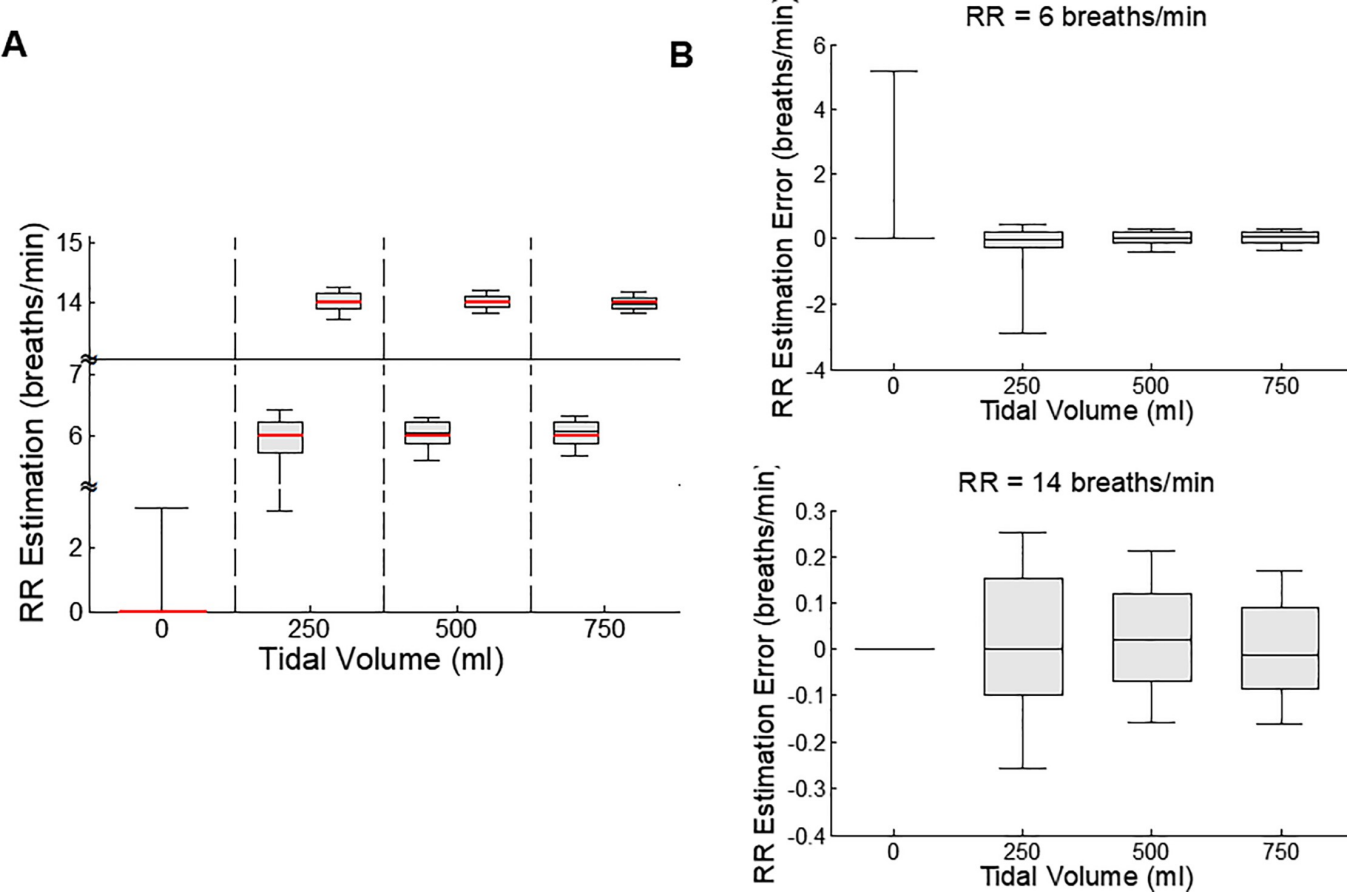
Respiratory rate (RR) estimations (A) and the associated estimation errors (B). The respiration rate was set to 6 or 14 breaths/min as marked by the thick red lines on (A). There were 9 records from 9 animals in case of 6 breaths/min, and 8 records from 8 animals in case of 14 breaths/min. In (A), the RR distributions between 0, 250, 500 and 750 ml (either at 6 or 14 breaths/min) were significantly different (p<0.001). There was statistical difference in the estimated RR distributions between any two 6 breaths/min (p<0.001, p<0.001, p = 0.112) or 14 breaths/min (p<0.056, p<0.05, and p<0.001), for any two TV settings. Each bar plot represents 90, 75, 50, 25 & 10% of all estimated values (A) or estimation errors (B).

The estimated RR values are displayed in Fig 4A. The median and standard deviation of the estimated RR values were 0±4.6, 6.0±2.1, 6.0±0.6 and 6.1±0.4 breaths/min at the 6 breaths/min RR settings, and 0±1.2, 14.0±0.2, 14.0±0.1 and 14.0±0.1 breaths/min at the 14 breaths/min RR settings, at TV of 0, 250, 500 and 750 ml, respectively. The RR distributions (Fig 4A) between 0, 250, 500 and 750 ml (either at 6 or 14 breaths/min) were significantly different (p<0.001). Among all estimated RR values, 97% exhibited errors of less than 1 breath/min. There was statistical difference in the estimated RR error distributions (Fig 4B) between any two 6 breath/min (p<0.001, p<0.001, p = 0.112) or 14 breaths/min (p<0.05, p<0.05, and p<0.001), for any two TV settings. Overall, these results demonstrate very robust and accurate RR estimations.

The TV and RR values estimated from the 51 episodes of apnea were 11.7±54.9 ml and 0±3.5 breaths/min, which were significantly smaller than other non-apneic periods. During these apnea periods, the time the RR estimation algorithm needed to detect apnea was 7.0±3.2 s. When we used 73 ml as the threshold for apnea detection, the time the TV algorithm needed to detect apnea was 8.6 ± 6.7 sec.

### Estimation of the respiratory rate from body surface signals

To determine the relationship of the number of ECG leads on the accuracy of the RR estimation, we estimated the RR by obtaining for each 32 beat sequence the ratio of any two body surface leads that provided an estimated error of less than 1 breath/min. In Table 1 we show the percent of RR estimations across all animals that resulted in an error of less than 1 breath/min for the different pairs of leads. In Table 1 we show the pair of leads percent of RR estimations across all animals that resulted in error of less than 1 breath/min. Pairing of ECGIII with V3 was the most commonly selected coupling, accounting for 91% of all estimations, while paring V3 with III and V2 with V5 and V2 with V1, resulted in 98.3% of the RR estimates to exhibit an error less than 1 breadth/min.

**Table 1:** Percent (% of errors smaller than the error limit at least one pair among the multiple pairs) of estimated respiration rates whose errors are smaller than 1 breath/min.

Number of Pairs	Numerator & Denominator	Percent
1	(V3,III)	91.1
2	(V3,III) (V2,V5)	96.6
3	(V3,III) (V2,V5) (V2,V1)	98.3

To further evaluate this method in accurately estimating the TV from a minimum number of ECG leads, we estimated the TV by obtaining the lead(s) that exhibited a percent error smaller than the error limit (105 ml, median error) in at least one lead. We observe that, at least in swine, all 12 leads are required to achieve a TV estimate less than 105 ml, 75% of the time.

## Discussion

The presence of SA has a significant negative impact on prognosis across many disease states but despite the availability of effective treatment, SA remains substantially underdiagnosed, and as a result, undertreated. To decrease the barrier to SA evaluation, we have developed a smartphone-based cardio-respiratory monitoring system, namely cvrPhone, that monitors RR and TV [17]. In this study, we tested the performance of the RR/TV estimation algorithms of the cvrPhone in diagnosing apnea. The results support that our algorithms can *first*, estimate the RR with an accuracy of 1 breath/min using only 2 ECG leads, ~91% of the time; *second*, estimate the TV with an accuracy of less than 105 ml using all 12 ECG leads, ~75% of the time; and *third*, detect apnea within ~7–8 seconds.

Inductive plethysmography (an inductive plethysmography sensor-band that consists of sinusoidal electrical wires and is excited by low amplitude, high frequency alternating current) and impedance pneumography (employing low amplitude, high frequency alternating current between two torso surface electrodes) are typical non-invasive methods of respiration monitoring in the sleep laboratory [18], [19]. These techniques evaluate respiration characteristics by measuring expansion and contraction of the rib cage (impedance pneumography) or both of the rib cage and abdomen. Recently, this methodology has been employed in smart garments, such as the LifeShirt (Vivometrics) and Hexoskin (Carre Technologies Inc), for the ambulatory monitoring of respiration. The validity and reliability of these wearable vests has been tested in diverse ambulatory conditions, including daily living activities, light to maximal excise, and patients with respiratory diseases [14, 15, 20–22]. In general, these studies showed acceptable validity and reliability for RR monitoring, but lower validity and reliability for TV and minute ventilation. Recently, researchers have constructed ambulatory impedance pneumography devices for quantitative monitoring of respiratory characteristics [13], and developed algorithms to remove motion artifact in impedance pneumography signals [23]. Further efforts have been made to develop new methods to estimate the RR from ECG and photoplethysmogram signals [24, 25]. However, the reported RR errors of these methods can be as high as 8–10%, while the TV could not be estimated. Wearable and smartphone based technologies have also been developed for non-invasive and continuous cardiac ambulatory monitoring using devices such as chest patches, necklaces, and smartwatches [9], [10], [12], [9].

In our study, during apnea imposed by a mechanical ventilator, the estimated TV and RR values were 11.7±54.9 ml and 0.0±3.5 breaths/min, which were significantly smaller than TV and RR estimation values during non-apnea settings. TV estimation values were statistically significantly different at different TV settings, and similarly were the RR estimations at different RR settings. These results suggest that our TV/RR estimation algorithms can be applied for SA detection, including Cheyne-Stokes respiration (CSR), whose breathing pattern is characterized by gradual increase and decrease of TV with periods of sleep apnea. The severity of sleep apnea is assessed by the apnea hypopnea index (AHI): the number of apneas and hypopneas per hour of sleep. The apnea/hypopnea in the index is defined as a cessation/reduction of breathing for 10 sec or more. In this study, the time our TV and RR algorithms needed to detect apnea was 8.6 ± 6.7 and 7.0±3.2 seconds, respectively. Our algorithms could be applied to estimate AHI from ECG signals. However, the apnea detection time requires further shortening to accurately measure AHI.

A potential limitation of this study, stems from the fact that the mean HR of the swine was ~100 bpm, while patients that suffer from sleep apnea tend to be bradycardic. High HRs facilitate the accurate estimation of the TV and RR during rapid changes of these signals. However, we have found that use of 16 beats to estimate the TV and RR are essentially no-different from the 32-beat, ones (data not shown). This finding permits that the TV and RR are estimated with the same accuracy during rapid changes in either the RR or TV, at HRs approximately half of those reported in this study, which would be close to those observed in patients with sleep apnea.

In conclusion, our ECG-derived respiration algorithms provide statistically meaningful TV estimations and accurate and precise RR estimations. In addition, both TV & RR estimation algorithms demonstrate reliable detection of apnea. These results suggest that our algorithms can be applied for SA detection in conjunction with ECG monitoring of ambulatory patients.
